# A Daily Diary Study on the Consequences of Networking on Employees' Career-Related Outcomes: The Mediating Role of Positive Affect

**DOI:** 10.3389/fpsyg.2018.02179

**Published:** 2018-11-13

**Authors:** Judith Volmer, Hans-Georg Wolff

**Affiliations:** ^1^Work and Organizational Psychology Group, University of Bamberg, Bamberg, Germany; ^2^Work and Organizational Psychology Group, University of Cologne, Cologne, Germany

**Keywords:** networking, diary study, conservation of resources theory, positive affect, career optimism, career satisfaction, job satisfaction, emotional exhaustion

## Abstract

Although researchers have shown that networking is positively associated with numerous long-term outcomes (e. g., salary, promotion) investigations of proximal outcomes of networking are still scarce. Building on Conservation of Resources theory (COR; Hobfoll, 2001, 2011) and conducting a daily diary study over five consecutive working days (*N* = 160 academics), we investigated short-term effects of networking on employees' career-related outcomes (i.e., career optimism and career satisfaction), job attitudes (i.e., job satisfaction), and well-being (i.e., emotional exhaustion). Further, we suggested that positive affect would act as a mediator. Results from hierarchical linear modeling (HLM) showed that daily networking relates to all four outcome variables. Moreover, positive affect mediated three of four hypothesized relationships, with a marginally significant effect for career satisfaction. By providing evidence for valuable short-term benefits of networking, our study extends existing research on positive long-term effects (for example on salary, promotions). Findings broaden the scope by integrating networking research with a positive organizational behavior perspective. We discuss practical implications with regard to career intervention strategies, study limitations, and prospects for future research.

## Introduction

Networking refers to goal-directed behavior aimed at creating, cultivating, and utilizing interpersonal contacts (Gibson et al., 2014). It is an important strategy in work and career self-management, because it enhances people's access to resources (Porter and Woo, 2015). Most prominently, scholars have adopted a boundaryless or protean career perspective (Arthur and Rousseau, 1996; Hall, 1996) to show that networking is positively related to career outcomes. These include, for instance, salary (Ng and Feldman, 2014a), promotions (Wolff and Moser, 2010), or career satisfaction (Ng and Feldman, 2014b). Further, networking is beneficial along the process of job search (e.g., Van Hoye et al., 2009). In addition, studies have utilized a social capital perspective to show that networking is positively related to measures of job performance (Blickle et al., 2012). In this vein, networking is also linked to measures of social capital, such as network size and structural holes (Wolff and Moser, 2006), and network size is further associated with the amount and diversity of information managers acquire from their contacts (Anderson, 2008).

While the literature clearly provides evidence for the value of networking regarding manifold outcomes, existing studies refer to more distal resources (i.e., long-term outcomes) and use a between-person perspective. Distal resources, for instance salary and promotions, typically accrue from proximal or short-term resources, such as strategic information or task advice from a broad range of contacts (Wolff et al., 2008). Distal outcomes thus accrue across several networking incidents. In line with this (implicit) cumulative assumption, the vast majority of studies have characterized networking in a time independent, dispositional manner. Networking questionnaires mostly ask people to assess how often they have shown networking behaviors in the past months or year (Forret and Dougherty, 2001). Using a between-persons perspective these studies show that networkers, that is, persons who consistently show networking behaviors (i.e., experience many networking incidents), acquire more resources than non-networkers.

We amend this perspective arguing that knowledge on short-term consequences of networking and how long-term outcomes accrue over networking events still remains limited. To our knowledge, only two studies have reported immediate and negative consequences of networking, showing that building contacts can provoke feelings of immorality (Casciaro et al., 2014) and that networking depletes self-regulatory resources (Wingender and Wolff, 2016). These studies have essentially adopted a focus on a single networking incident and report negative consequences. Thus, there exists some tension between immediate negative and long term positive outcomes and there might be a tipping point, where experiences around networking incidents change. Therefore, we focus on an intermediate, but still short time frame and examine networking on a daily level. We posit that negative consequence are limited to instantaneous and short-lasting processes (Tyler and Burns, 2008), but, at the end of the day, the anticipation or acquisition of resources yields positive outcomes for individuals, as well. Our within-person approach responds to claims that career success is an emergent process evolving as a function of time and experience (Heslin and Turban, 2016).

In the present study, we examine short-term consequences of networking incidents at the day level by using a diary study as recently suggested by Kalish et al. (2015). Our main research question is thus how variations in daily networking activities affect people's experiences at work and after work. More specifically, we adopt Conservation of Resource theory (COR; Hobfoll, 2001, 2011) as a broad framework of stress and motivation. COR is useful in explaining resource gains from networking as it has been adopted to also explain resource gains in the workplace (Gorgievski et al., 2011). Based on the notion that people must invest resources to gain resources, we argue that daily networking activities afford the acquisition of resources and lead to positive psychological and attitudinal consequences. Specifically, we examine whether daily networking activities result in increased daily job and career satisfaction as well as career optimism because these variables have been identified as important constructs regarding COR research (e.g., Harvey et al., 2007). Based upon COR theory's premise that resource gain attenuates burnout, we also examine whether networking is associated with reduced emotional exhaustion, which is a well-researched dimension of burnout in resource based theories (Bakker et al., 2014, p. 400; Gorgievski and Hobfoll, 2008, p. 2). Finally, to explain how networking is linked to our outcomes (i.e., emotional exhaustion, job satisfaction, career optimism, and career satisfaction), we refer to broaden-and-build theory (Fredrickson, 2001) by examining networking activities as a correlate of positive affect at work and thus suggest positive affect to act as a mediator (Xanthopoulou et al., 2012).

The present study contributes to our knowledge on networking and resources in at least two ways. First, this is the first study to examine daily fluctuations of networking and its consequences, thereby using a within-person perspective. Ignoring whether individuals are frequent networkers or not, the unit of analysis is daily networking activities which may vary across individuals, but also within days of the same individual. We thus provide further insights into daily processes of resource acquisition by means of networking activities and contribute to the very few studies relating work experiences to career outcomes on a daily level (e.g., Zacher, 2015). Second, we provide a further outline on resources available from networking based upon COR theory. Although access to resources is a central tenet in the networking literature, the term is not well-developed and COR theory provides a suitable framework to organize resources. We also expand upon the positive consequences of networking by showing that it also yields psychological benefits (e.g., career optimism, career satisfaction) on a daily level. In sum, our focus on resource gains further adds to the growing literature that utilizes COR theory to theorize on positive consequences of resource acquisition (Quinn et al., 2012; van Woerkom et al., 2016) and the positive organizational behavior perspective (Quick et al., 2010).

## Theoretical background

### Networking

Networking refers to a diverse set of behaviors aimed at building, maintaining, and using work related contacts. Networking is not a stable personality trait, but a behavior syndrome, that is, a set of interrelated behaviors consistently shown by people (Wolff et al., 2008). Examples of networking behaviors are going out with others for drinks after work (Forret and Dougherty, 2001), forming alliances with people in other units, offering help to others in work related matters, or exchanging useful information and gossip (Michael and Yukl, 1993). Moreover, networking is distinct from social capital, which refers to a structural level of analysis and focuses on network characteristics, such as network size or network density. In contrast to social capital, networking focuses on individual behaviors and can thus be considered as an antecedent of social capital or as a part of the “engine of action” (Coleman, 1988, p. S96) that is missing from theories on social capital. However, despite the fact that the two concepts refer to different levels of analysis, they are related to each other (Gibson et al., 2014) which is why we will at times rely on the social capital literature.

### Networking and resources

According to Porter and Woo (2015), all scholars emphasize that networking is a means to obtain access to resources. Moreover, it refers to an exchange of resources (Michael and Yukl, 1993; Porter and Woo, 2015) that is governed by norms of reciprocity. In this vein, networking also involves providing others with resources and a temporal perspective where provision and reception of resources might not occur simultaneously but at different points in time (Coleman, 1988). The proverbial owing of a favor implies that people will keep track of what they give and receive from a contact and that considerations of reciprocity play an important role in the decision to provide resources. Due to the temporal separation between resource exchanges, trust that others will return favors plays an important role with regard to networking relations (Michael and Yukl, 1993).

Regarding resources, lists from the literature include salary, promotions, and career satisfaction (Forret and Dougherty, 2004), job performance (Blickle et al., 2012), or strategic information, task advice, friendship, and buy-in (Podolny and Baron, 1997). Porter and Woo (2015) arrayed these resources based upon Foa and Foa's (1980) particularistic-universalistic dimension that ranges from friendship at the particularistic end to money at the universalistic end, with resources such as status, information, or goods in between. In a further exploration of networking resources, Wolff et al. (2008) distinguished *distal resources*, such as career success or power that people accrue from their network over time from *proximal resources* (e.g., task advice, strategic information) that are available from a single networking contact. Here, we use this distinction to further delineate the relation between networking and resources in the subsequent section. Wolff et al. (2008) suggest that distal resources are usually the result of the acquisition of proximal resources. For example, people combine information from different contacts into an innovative idea. They may also acquire information on the requirements for a potential promotion from one contact while using another contact to get access to those persons who are in charge of awarding the respective promotion.

### Networking and conservation of resources theory

COR theory defines resources broadly as entities that people value. Hobfoll (2001) has suggested classifying resources into several broad categories: objects/conditions, social support, energies, and constructive resources. These resource categories can be arranged along two dimensions which refer to their respective source (i.e., the context vs. the person) and transience (i.e., durable or structural vs. transient or volatile; see also ten Brummelhuis and Bakker, 2012). First, *objects or conditions* refer to durable resources which are located in the context, for example employment, having a social network, or being married. Second, *social support* is a resource broadly referring to instrumental and emotional support from others. It is also located in the context (i.e., other persons), but is more transient than objects or conditions. Third, *energies* are transient resources that lie within a person. Examples are mood, time, or physical and cognitive energy. Finally, durable resources that have their origin within persons fall into the category of *constructive resources*. Examples are people's skills, knowledge, or experience. In addition, some constructive resources represent *key resources*, because they refer to stable individual differences which can be broadly applied in the management of stressful circumstances. For this reason, they are of higher importance than other resources. Examples for key resources are self-efficacy, optimism, or self-esteem (Hobfoll, 2002; ten Brummelhuis and Bakker, 2012). Note that Hobfoll further considers macro resources, such as culture or public policies which we do not discuss further as they are not relevant regarding the context of the present study.

With COR theory's recent extension to resource gains in the workplace (Gorgievski and Hobfoll, 2008; ten Brummelhuis and Bakker, 2012), it provides a convenient scheme to organize the variety of resources listed in the networking literature and further delineates the gain processes associated with networking. First, networking is an important skill and thus represents a constructive resource. People invest time and energy (i.e., transient energy resources) into networking in order to gain other resources (Wingender and Wolff, 2016). The display of networking behavior thus entails both, the utilization of energy resources and the usage of constructive resources to accumulate further resources. Second, COR theory offers a classification of those resources that can be gained through networking and highlights that the literature has focused on the premise that networking mainly yields contextual resources. Specifically, people utilize networking to obtain the transient resource of social support (e.g., task advice, strategic information), which in turn can result in the acquisition of more durable resources (mostly conditions; e.g., career success). In this vein, social support and durable resources closely resemble Wolff et al.'s (2008) distinction of proximal and distal resources. Proximal resources (i.e., social support) are typically available from a single contact, whereas distal resources (i.e., conditions) are typically the accumulated result of resources from an entire network of contacts (e.g., social capital, career success). In sum, networking plays an important role in resource caravans which refers to the phenomenon that resources are typically linked to other resources and tend to aggregate (Hobfoll, 2001). Note however, that additional gain processes are possible as not only volatile resources can yield durable resources (e.g., strategic information increases the likelihood of promotion), but also vice versa (e.g., social capital yields facilitated access to task advice).

COR theory further links resource gain to important additional outcomes (Gorgievski and Hobfoll, 2008). More specifically, ten Brummelhuis and Bakker (2012) have recently suggested the distinction of production outcomes (e.g., completing tasks, service quality), behavioral outcomes (e.g., absenteeism, availability at home), and attitudinal outcomes, such as satisfaction, commitment, or well-being. The link between resource gains and outcomes is also an explanation for the link between networking and outcomes: Networking, as an important component of resource gain processes leads to job performance (i.e., a production outcome), satisfaction, and work engagement (attitudinal outcomes). Similarly, as burnout also results from a lack of resources (Gorgievski and Hobfoll, 2008), resource acquisition by means of networking counteracts exhaustion.

In the literature, these processes and the relationship between networking and psychological outcomes have not been examined on this finer grained level. Many studies provide evidence for long-term beneficial effects of networking and thus the relationship between networking and conditions (e.g., career success, Forret and Dougherty, 2004; Wolff and Moser, 2009). There is also some evidence for the core assumption of the networking literature that networking yields social support, such as task advice or information (Burke, 1984). Yet, even these short-term processes have been examined by means of between-person designs which utilize broad time frames to relate networking and resources (e.g., within the past year, cf. Forret and Dougherty, 2001). Note however that these processes occur within persons and between-person designs rely on strong assumptions to infer these processes (Ohly et al., 2010). Therefore, within-person studies are necessary to examine the short-term effects of networking.

### Development of hypotheses

**Figure 1:**
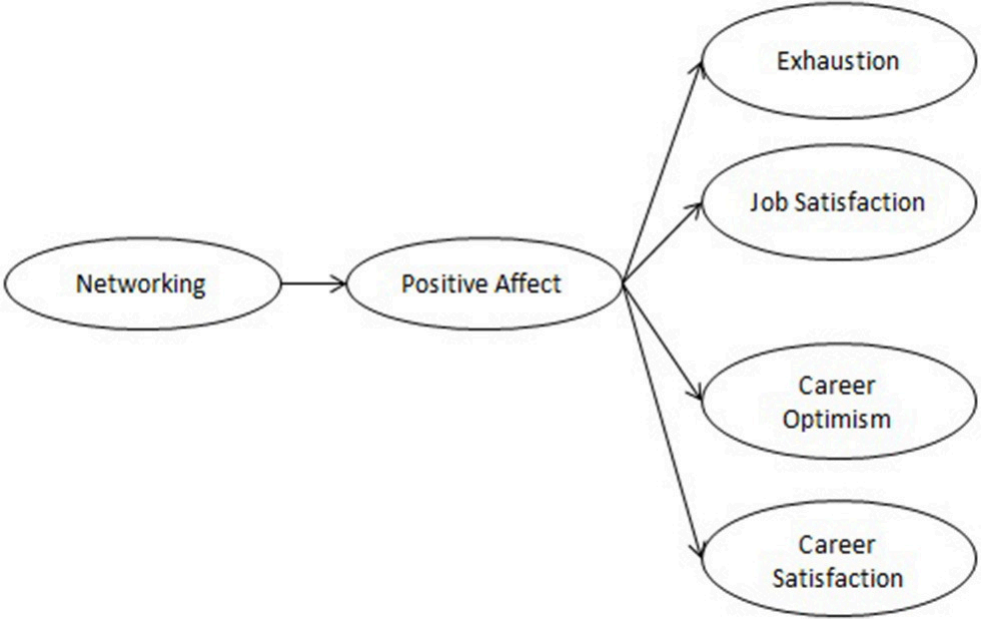
Conceptual model of the effects of daily networking (within-person model, between-person controls omitted).

In the present study, we examine within-person fluctuations of networking to capture the processes by which networking affects resource acquisition on a daily level. Figure 1 provides an overview of our conceptual model. Note that, as our theorizing refers to within-person processes, all constructs in Figure 1 refer to the within-person level. In contrast to prior research which has predominantly focused on contextual resources (e.g., task advice, career success) we focus on people's affective and cognitive reactions to networking across the day. Specifically, we investigate emotional exhaustion and job satisfaction. In addition, we examine two immediate career-related outcomes, career optimism and career satisfaction. We chose these constructs for two reasons: First, assessing resources acquired by networking on a daily level are too idiosyncratic. For instance, a list by Burke (1984) consists of 21 resources and is probably not exhaustive. In accordance, Halbesleben et al. (2014) state that resources might differ across occupations and result from using contacts to reduce workload, to solve a problem, or talking to managers about a promotion, and they also include securing future access to resources by means of building or fostering contacts. They also propose to assess outcomes as indicators for changes in resources as a remedy. Following this advice, we opted to focus on a broad range of outcomes of resource gain processes. Second, the outcomes we examine cover a broad range of theoretical predictions from the networking and COR literatures. In particular, using job as well as career related variables is in accordance with the proposition that networking assists people in their work or career (Forret and Dougherty, 2004). Moreover, these constructs cover attitudinal outcomes (i.e., satisfaction), well-being (i.e., exhaustion), and a key resource (i.e., optimism).

#### Emotional exhaustion

Emotional exhaustion refers to the stress dimension of burnout and includes feelings of being exhausted by the emotional demands of an individual's work (Maslach et al., 2001). Emotional exhaustion has already been shown to fluctuate on a daily basis (e.g., Barling and MacIntyre, 1993; Demerouti et al., 2004; Simbula, 2010; Hülsheger et al., 2013). For example, Simbula (2010) found in a diary study over five consecutive working days that 39% of the variance in emotional exhaustion was at the within-person level. Likewise, Blanco-Donoso et al. (2017) found in a 5-day study that 30% of the variance of emotional exhaustion was at the within-person level, and Aldrup et al. (2017) found in a 10-day study that a percentage of 69% of the variance of emotional exhaustion can be accounted to the within-person level.

As described by Aldrup et al. (2017), there is a growing number of research that builds on the variability approach of stressors and resources (e.g., Kitching, 2009; Bakker and Bal, 2010; Simbula, 2010; cf. Aldrup et al., 2017, p. 22). Aldrup et al. (2017) further describe that positive and negative daily events can either promote or harm employees' daily well-being.

Drawing on COR theory, resource loss or lack of resources can lead to burnout. Because networking is a means to acquire resources, it should counteract net losses of resources and thus feelings of exhaustion. For instance, getting advice from a colleague on a statistical problem can save extensive reading on the problem and can thus help to conserve resources, or a colleague promising to put in a word for a promotion heightens a person's mood (i.e., an energy resource). In a similar vein, studies have shown that (work-related) interpersonal OCBs reduce exhaustion (e.g., Koopman et al., 2016). Thus, we assume that employees with a high level of daily networking experience less emotional exhaustion. Note that we do acknowledge that networking also requires the investment of resources and can lead to immediate resource depletion (Wingender and Wolff, 2016). Yet, these effects are expected to be of short duration (Tyler and Burns, 2008) and should not play a role in day-level experiences. In addition, COR theory holds that people invest resources in hope to gain further resources or minimize losses (i.e., an assumption of rational choice). In line with COR theory we therefore assume that people invest energy into networking only when they anticipate that it pays off. In other words, even though networking entails resource investments that potentially lead to exhaustion, these effects are usually offset by resource gains attained from networking.

**Hypothesis 1**. Daily networking at work will be negatively related to daily emotional exhaustion.

#### Job satisfaction

Job satisfaction refers to an individual's evaluation of his or her job or job facets, such as job tasks, relationships to coworkers, opportunities for advancement, pay, or recognition and appreciation (Spector, 1985). Satisfaction also represents an important attitudinal outcome of resource acquisition (ten Brummelhuis and Bakker, 2012). We propose that daily networking activities should boost job satisfaction by two means. First, given that networking is a means to acquire work-related resources (e.g., task advice), higher resource availability in the job should result in higher job performance and thus recognition and appreciation. Second, networking refers to cooperative social interactions. Because job satisfaction also entails evaluations of the social environment at work, experiencing positive relationships with others at work raises evaluations of the social facets of job satisfaction (e.g., satisfaction with coworkers and supervisors, see also Koopman et al., 2016).

**Hypothesis 2**. Daily networking at work will be positively related to daily job satisfaction.

#### Career optimism

Career optimism has originally been defined as “a disposition to expect the best possible outcome or to emphasize the most positive aspects of one's future career development, and comfort in performing career planning tasks” (Rottinghaus et al., 2005, p.11). It has been developed on the basis of dispositional optimism (Scheier et al., 2001; Seligman, 2008). According to COR theory, optimism is a key resource that broadly facilitates the attainment of other resources. In the present study, we use the more specific construct of career optimism, because it closely corresponds with the predicted work or career related effects of networking behaviors. On a between-person level, career optimism is associated with a variety of objective and subjective career indicators, such as salary, promotions, career satisfaction, and employability (Rottinghaus et al., 2005; Spurk and Volmer, 2013).

Recently, scholars have gone beyond the trait perspective and found evidence for optimism as a malleable state and suggested that coaching or encouragement foster optimism (Kluemper et al., 2009; Xanthopoulou et al., 2012). For example, using a diary study, Xanthopoulou et al. (2009) showed that daily fluctuations in job resources (e.g., autonomy, team climate) affected daily fluctuations in optimism. As networking assists people in the acquisition of actual (e.g., background information on a job vacancy) or potential contextual resources (e.g., building contacts to important stakeholders), we predict that this experience will also lead to higher expectations for a person's future career.

**Hypothesis 3**. Daily networking at work will be positively related to daily career optimism.

#### Career satisfaction

Career satisfaction is an important topic in organizational research, because subjective feelings of success are related to many facets of work behavior and well-being (e.g., Ng et al., 2005; Abele and Spurk, 2009). Career satisfaction as individuals' idiosyncratic evaluations of their own careers has been shown to be one central indicator of subjective career success (Boudreau and Boswell, 2001; Ng et al., 2005; Abele et al., 2011). It is the evaluation of an individual's progress toward meeting different career-related goals (e.g., income, achievement, development) and career-related successes (e.g., overall career success; see also Hofmans et al., 2008). According to a meta-analysis of between-person effects conducted by Ng and Feldman (2014b), networking is related to higher career satisfaction, because it provides access to important career related outcomes. For example, Wolff and Moser (2010) found that building internal or external contacts is a predictor for internal promotions or employer changes, respectively. With regard to the present study's day-level design, we follow Zacher (2015) who found evidence for meaningful day-level variation in career satisfaction, arguing that daily variation in career adaptability affects daily career satisfaction. We suggest that daily fluctuations in networking are associated with the promotion of important contacts or attainment of career related information. In addition, networking interactions yield feedback or appraisals of a person's accomplishments (e.g., receiving compliments on a paper in an A-Journal) which enhances subjective evaluations (e.g., perceived marketability, cf. Porter and Woo, 2015) of career progress.

**Hypothesis 4**. Daily networking at work will be positively related to daily career satisfaction.

#### Positive affect

Positive affect (PA) is defined as a state that comprises being “enthusiastic, active, and alert” (Watson et al., 1988, p. 1063). On the basis of COR theory (Hobfoll, 2001, 2011), we regard networking as a means to proactively acquire valuable resources at work. Halbesleben et al. (2014) suggest that resource gains should also have an impact on affective states and also ten Brummelhuis and Bakker (2012) postulate that receiving support increases mood. Likewise, we assume that networking is a pleasant experience because it yields actual resources or the potential to receive them in the future. This is in line with findings from diary studies in other areas that found supportive online interactions (Oh et al., 2014) as well as interpersonal organizational citizenship behavior (Koopman et al., 2016) to enhance PA. Further, PA enhanced the sense of community and life/job satisfaction (Oh et al., 2014).

Similar to previous research (Xanthopoulou et al., 2012) and building on broaden-and-build theory (Fredrickson, 2003) we suggest that positive affect has an immediate (i.e., daily) effect on employees' career-related evaluations as well as their well-being. According to the broaden-and build theory, the experience of positive emotions broadens people's thought-action repertoires, which in turn helps to build personal resources. Applied to our research question, people should feel less exhausted on days with a high level of networking because networking uplifts their mood which in turn—as personal resources are built—diminishes the level of experienced daily exhaustion. Further, employees should report a higher level of daily job satisfaction, and their daily career optimism and daily career satisfaction should be boosted from their positive mood which in turn is triggered by their respective networking behavior. Accordingly, meta-analytic findings show significant associations between PA and emotional exhaustion and job satisfaction (*ρ* = −0.32 and *ρ* = 0.34, respectively; Thoresen et al., 2003). Regarding career optimism, there is also evidence for a positive association with PA (*r* = 0.32, *p* < 0.001; Rottinghaus et al., 2005).

Taken together, we propose that PA mediates the relationships between daily networking behavior and employees' short-term reactions, including career-related outcomes (i.e., career optimism and career satisfaction), job attitude (i.e. job satisfaction), and well-being (i.e., emotional exhaustion).

**Hypothesis 5**. State PA will mediate the relationship between daily networking at work and (a) daily emotional exhaustion, (b) daily job satisfaction, (c) daily career optimism, and (d) daily career satisfaction.

## Methods

### Overview

We used a within-person design and conducted an experience sampling study with 160 German academic staff members from four different German universities. Data were collected with a general survey and with daily surveys. Participants filled in daily surveys over five consecutive workdays (Monday–Friday), two times per day (i.e., at the end of the workday and at bedtime). The general survey was completed 1 week before the start of the daily surveys. We collected data on networking and positive affect at the end of the workday and data on emotional exhaustion, job satisfaction, career optimism, and career satisfaction at bedtime.

### Procedure and sample

We approached academic staff (i.e., researchers, except tenured professors) at four German universities via e-mail, described the aim of the study, and asked for participation. We introduced our study as research on networking. When interested, participants could send an e-mail or fax to the research team, indicating their interest of participation in the study. After registration, participants received a survey package with detailed instructions on when and how to fill out the questionnaires. Participants were informed on the first page of the survey that data were collected anonymously and that data would be treated confidentially. Upon agreement, participants filled in the general questionnaire and one week later they filled in the daily surveys over five consecutive workdays. To match questionnaires while assuring confidentiality, participants were asked to indicate a code on each questionnaire. As incentives, we offered participation in a lottery and detailed feedback on the results after study completion.

We chose German academia as our research context because the German system of higher education is very competitive and exhibits many characteristics that require researchers below the professor's level to manage boundaryless careers (Kreckel, 2017). In fact, academia in Germany shares many characteristics with the *baseball team* career system described by Sonnenfeld and Peiperl (1988). This system is often employed in industries such as for example advertising, consulting, investment banks, or broadcasting. It is characterized by a lack of employment security, pressure for creativity, and an orientation to external labor markets. In Germany, people at the doctoral and postdoctoral level are usually employed by universities or receive an equivalent scholarship. Comparable to many consulting firms, universities have an “up or out” and “bottleneck” system with many positions at the doctoral level, fewer positions at the postdoctoral, and even less at the professor's level. Moreover, legislation largely limits employment below the professor's position to a maximum of 12 years and there are only very few permanent positions for postdocs within the German academic system. In addition, usually a person can only attain a professor's position at his or her current institution, when they have worked for other employers, that is, they are required to have changed their employer at least once—or they can only get a professors position at another institution. Whereas, doctoral and postdoctoral employees might reflect junior and senior consultants, professors are more similar to partners in consultancy as professors usually get tenure immediately in Germany. Thus, while doctoral and postdoctoral employees are pushed to advance their scientific career across institutional boundaries in a competitive environment, incentives for professors to advance their career are most likely pull factors, such as reputation of an institution or resources for research. Due to the differences of professors compared to researchers at the doctoral or postdoctoral level, we excluded professors from our sample.

Overall, 174 employees agreed to participate in our study. From these, 160 sent back questionnaires to our research team, resulting in a response rate of 91.95%. Participants (58% were women) had an average age of 32.39 years (*SD* = 6.27) and on average 5.78 years (*SD* = 5.82) of research job experience. Most participants held a master's degree (64%), followed by a Ph.D. (29%), and a habilitation (a post-Ph.D. qualification necessary for professorships, 6%). Participants were employed within different academic fields: social and economic sciences (30%), mathematics and natural sciences (29%), engineering sciences (12%), linguistic and cultural studies (12%), and law (2%). Fifteen percent of the participants reported to work in other academic fields.

### Measures

#### General measures

In the general survey, we measured trait networking and trait positive affect as well as demographic variables (i.e., age, gender, tenure, and education).

##### Trait networking

To measure *trait networking*, we used a scale developed by Wolff and Moser (2006) which consists of six subscales with each of them referring to different types of networking behaviors: (1) building internal contacts (six items, e.g., “I use company events to make new contacts”), (2) maintaining internal contacts (seven items, e.g., “I catch up with colleagues from other departments about what they are working on”), (3) using internal contacts (eight items, e.g., “I use my contacts with colleagues in other departments in order to get confidential advice in business matters”), (4) building external contacts (seven items, e.g., “I accept invitations to official functions or festivities out of professional interest”), (5) maintaining external contacts (seven items, e.g., “I ask others to give my regards to business acquaintances outside of our company”), and (6) using external contacts (eight items, e.g., “I exchange professional tips and hints with acquaintances from other organizations”). Participants rated on a 4-point Likert type scale (1 = *never*, 4 = *always*) how often they had displayed different networking behaviors in the past 6 months. Cronbach's alpha for the overall networking scale was 0.93.

##### Trait positive affect

We used the German translation of the Positive and Negative Affect Schedule (PANAS; Watson et al., 1988) by Krohne et al. (1996) to assess dispositional *positive affect*. Participants assessed six positive adjectives (e.g., interested, active) on a 5-point scale ranging from 1 (*not at all*) to 5 (*entirely*), indicating to what extent each adjective reflected how they felt in general. Cronbach's alpha was 0.67.

##### Control variables

We controlled for several variables to exclude alternative explanations for our findings (e.g., Becker, 2005). First, we controlled for gender (1 = *men*, 2 = *women*), because differential relationships of networking with resources have been reported (Forret and Dougherty, 2004), and Halbesleben cautioned to “account for the role of gender when exploring the relationships between support and burnout” (Halbesleben, 2006, p. 1142). We also controlled for age and tenure as both reflect experience that might affect networking and our outcomes. For example Wolff et al. (2008) report that networking might decline with age and building contacts should be more likely for newcomers. Likewise career goals and experiences change, when people begin the doctorate as compared to its completion or when they try to get established in their field of research in the postdoc stage. In this vein, we also controlled for education (1 = *bachelor degree*, 2 = *master degree*, 3 = *Ph.D*., 4 = *habilitation*), which has also been shown to be positively related to networking (Wolff et al., 2008). As we were interested in daily fluctuations, we also controlled for trait networking using Wolff and Moser's 44-item measure (2006b; α = 0.91) and trait positive affect (α = 0.84) using the PANAS (Watson et al., 1988). All control variables were assessed in the general survey.

#### Daily measures

In order to temporally separate antecedents (i.e., networking) and outcomes (e.g., exhaustion, career optimism), we assessed networking and PA at the end of work and daily attitudinal and well-being outcomes (i.e., emotional exhaustion, job satisfaction) as well as career-related outcomes (i.e., career optimism, career satisfaction) at bedtime.

##### Networking

*Networking* was assessed with eight items adapted fromWolff and Moser's (2006) networking scale to fit the day-level assessment of networking. To include the full breadth of networking, items covered building (e.g., “Today, I actively engaged in building or deepening new contacts”), maintaining (e.g., “Today, I tended to maintain my contacts”), and using contacts (e.g., “Today, my contacts were beneficial concerning business matters”). In contrast to the assessment of trait networking, we did not distinguish between internal and external networking in the daily questionnaires as we aimed at reducing the burden for the participants (Ohly et al., 2010). For the respective day, participants indicated whether they performed these networking behaviors (1 = *not at all*, 6 = *to a very high extent*). Cronbach's alpha ranged from 0.83 to 0.95 over the 5 days (mean α = 0.91).

##### Emotional exhaustion

*Daily emotional exhaustion* was measured at the end of the workday with eight items from the Oldenburg Burnout Inventory (OLBI; Demerouti et al., 2003). Participants indicated their agreement on 4-point Likert-scales (1 = *totally disagree*, 4 = *totally agree*). A sample item was “Today, I felt emotionally exhausted at work.” Cronbach's alpha ranged from 0.81 to 0.83 over the 5 days (mean α = 0.82).

##### Job satisfaction

Following Kunin (1955), a series of seven faces, which show feelings from extremely negative (1 = *totally dissatisfied*) to extremely positive (7 = *totally satisfied*), were used to assess how satisfied participants were with their job at the moment. We used this single-item measure to assess job satisfaction for three reasons: First, meta-analytic work by Wanous et al. (1997) demonstrated that overall job satisfaction correlates highly with multiple-item measures (corrected *R* = 0.67), thus providing an efficient alternative to more comprehensive facet measures of job satisfaction. Second, Ironson et al. (1989) further argued that a single item captures the essence of job satisfaction better than a more specific subscale measure. Third, meta-analytical work by Kaplan et al. (2009) showed that Kunin's faces scale is best suited to capture both employees' affective and cognitive reactions to work. Thus, a single-item faces scale is well-suited to obtain a comprehensive rating of employees' attitude to work.

##### Career optimism

We measured *career optimism* with three items from the Career Futures Inventory (CFI; Rottinghaus et al., 2005) with a German version developed by Spurk and Volmer (2013). A sample item was “Today, I was totally amazed by thinking about my career future.” Participants rated the extent of agreement with each item on a 5-point Likert scale (1 = *totally disagree*, 5 = *totally agree*). Cronbach's alpha ranged from 0.84 to 0.90 over the 5 days (mean α = 0.87).

##### Career satisfaction

We assessed *career satisfaction* with three items from the Career Satisfaction Scale (CSS; Greenhaus et al., 1990) with a German version from Wolff and Moser (2009). Participants were instructed to rate their day-level evaluation of career satisfaction on 5-point Likert scales (1 = *totally disagree*, 5 = *totally agree*). A sample item was “Today, I am satisfied with the achievements that I have reached during my career.” Cronbach's alpha ranged from 0.81 to 0.94 over the 5 days (mean α = 0.87).

##### Positive affect

Following Sonnentag et al. (2008), we assessed *daily positive affect* with six items from the Positive and Negative Affect Scale (PANAS; Watson et al., 1988). On 5-point Likert scales (1 = *not at all*, 5 = *entirely*), employees rated the intensity of their momentary affective experience of emotional states described by adjectives such as active, interested, and excited. Cronbach's alpha ranged from 0.82 to 0.87 over the 5 days (mean α = 0.84).

## Results

We analyzed our data with a multilevel random coefficient model using hierarchical linear modeling (HLM Version 7; Raudenbush et al., 2011). Level 2 data were centered on the grand mean and Level 1 data were centered on the respective person mean. In order to test whether HLM analyses were appropriate, we examined the within-person and between-person variance components of our study variables (i.e., networking, positive affect, emotional exhaustion, job satisfaction, career optimism, and career satisfaction). Regarding daily networking, the intra-class correlation indicated that 70.14% of the variance was at the individual level. Regarding daily positive affect, within-person variance was 52.04%. Moreover, 65.28% of the variance in daily emotional exhaustion, 28.17% of the variance in daily job satisfaction, 39.66% of the variance in daily career optimism, and 35.34% of the variance in daily career satisfaction were at the individual level. Thus, while ICCs had quite a large range (i.e., 0.28 < ICC < 0.70), all variance components were significant at *p* < 0.01 (cf. Tables **2**–**5**). More importantly, their absolute size was well above the minimum scholars have labeled substantial (Ilies et al., 2007 ICC > 0.20; Smet et al., 2016 ICC > 0.10), justifying running HLM analyses. We used the restricted maximum likelihood procedure in HLM to test our hypotheses. All hypotheses were tested in a two-tailed way. Means, standard deviations, and zero-order correlations of study variables are presented in Table 1.

**Table 1:** Means, standard deviations of variables in the multilevel model, and correlations among person-level variables and among day-level variables.

**Variable**	***M***	***SD***	**1**	**2**	**3**	**4**	**5**	**6**	**7**	**8**	**9**	**10**	**11**	**12**
**PERSON-LEVEL MEASURES**^a^														
1.Age	32.40	6.27											
2.Gender^b^	1.58	0.49	−0.11										
3.Tenure	5.78	5.82	0.85**	−0.08									
4.Education^c^	2.38	0.63	0.60**	−0.01	0.70**								
5.Trait networking	2.40	0.46	−0.04	−0.17*	0.00	0.04	(0.93)						
6.Trait positive affect	3.67	0.52	0.08	0.02	0.07	0.15	0.17*	(0.67)					
**DAILY MEASURES**^d^														
7.Networking	2.15	1.16							(0.91)				
8.Positive affect	2.78	0.80							0.13**	(0.84)			
9. Emotional exhaustion	2.17	0.62							0.03	−0.43**	(0.82)		
10. Job satisfaction	5.16	1.61							0.04	0.32**	−0.55**	–	
11. Career optimism	3.22	0.92							0.05	0.40**	−0.33**	0.50**	(0.87)
12. Career satisfaction	3.44	0.92							0.05	0.25**	−0.27**	0.44**	0.64**	(0.87)

*Cronbach's alphas are listed on the diagonal. Cronbach's alphas of the daily measures are mean alphas over 5 days*.

a *N = 160*.

b *Gender (1 = male; 2 = female)*.

c *Education (1 = bachelor degree; 2 = master degree; 3 = Ph.D.; 4 = habilitation)*.

d *n = 463–587*.

* *p < 0.05*.

** *p < 0.01*.

### Daily networking and attitudinal/well-being outcomes

#### Emotional exhaustion

In Hypothesis 1, we predicted that day-level networking would be negatively related to emotional exhaustion at bedtime. Results are shown in Table 2. In the Null model, the intercept was the only predictor. In Model 1, we added the control variables (i.e., age, gender, tenure, education, trait networking, and trait positive affect). Analyses showed that Model 1, in which the control variables were entered, did not show an improvement over the null model (Δ − 2 × log = 6.31, ns). Model 2, which also included networking, showed a significant improvement over Model 1 (Δ − 2 × log = 89.60, *p* < 0.01). Analyses indicated that day-level networking was negatively related to emotional exhaustion at bedtime (γ = −0.07; *SE* = 0.03, *t* = −2.23, *p* < 0.05). Thus, we found support for Hypothesis 1.

**Table 2:** multilevel estimates for models predicting day-specific emotional exhaustion at bedtime.

**Variable**	**Null model**			**Model 1**			**Model 2**			**Model 3**		
	**Estimate**	***SE***	***t***	**Estimate**	***SE***	***t***	**Estimate**	***SE***	***t***	**Estimate**	***SE***	***t***
Intercept	2.18	0.04	58.03**	2.18	0.04	61.27**	2.18	0.04	57.53**	2.18	0.04	57.59**
Age				−0.02	0.01	−2.17*	−0.02	0.01	−2.02*	−0.02	0.01	−2.04*
Gender^a^				0.03	0.07	0.36	0.05	0.08	0.57	0.04	0.08	0.51
Tenure				0.00	0.01	0.21	0.00	0.01	0.18	0.00	0.01	0.09
Education^b^				−0.00	0.08	−0.03	−0.03	0.08	−0.33	−0.02	0.08	−0.20
Trait networking				0.18	0.08	2.27*	0.20	0.08	2.43*	0.21	0.08	2.45*
Trait positive affect				−0.14	0.07	−1.94	−0.13	0.08	−1.61	−0.12	0.08	−1.59
Networking							−0.07	0.03	−2.23*		
Positive affect										−0.37	0.05	−7.13**
Δ Deviance					6.31			89.60**			
Level 1 intercept		0.4995			0.4993			0.4925			0.4252
Level 2 intercept		0.3643			0.3333			0.3417			0.3688

a *Gender (male = 1; female = 2)*.

b *Education (1 = bachelor degree; 2 = master degree; 3 = Ph.D.; 4 = habilitation)*.

* *p < 0.05*.

** *p < 0.01*.

#### Job satisfaction

In Hypothesis 2, we predicted that day-level networking would be positively related to job satisfaction at bedtime. Results are shown in Table 3. Analyses showed that Model 1, in which the control variables were entered, did not show an improvement over the null model (Δ −2 × log = 0.96, ns). Model 2, which also included networking, showed a significant improvement over Model 1 (Δ −2 × log = 198.69, *p* < 0.01). Analyses indicated that day-level networking was positively related to job satisfaction at bedtime (γ = 0.15; *SE* = 0.05, *t* = 3.17, *p* < 0.01), yielding support for Hypothesis 2.

**Table 3:** Multilevel estimates for models predicting day-specific job satisfaction at bedtime.

**Variable**	**Null model**			**Model 1**			**Model 2**			**Model 3**		
	**Estimate**	***SE***	***t***	**Estimate**	***SE***	***t***	**Estimate**	***SE***	***t***	**Estimate**	***SE***	***t***
Intercept	5.17	0.12	43.24**	5.16	0.12	44.45**	5.18	0.12	42.69**	5.18	0.12	42.70**
Age				0.02	0.04	0.56	0.02	0.04	0.55	0.02	0.04	0.57
Gender^a^				0.02	0.24	0.10	−0.05	0.25	−0.21	−0.00	0.25	−0.00
Tenure				0.00	0.04	0.02	0.01	0.04	0.19	0.01	0.04	0.24
Education^b^				0.15	0.26	0.56	0.15	0.27	0.56	0.13	0.27	0.49
Trait networking				−0.47	0.26	−1.83	−0.46	0.27	−1.68	−0.47	0.27	−1.72
Trait positive affect				0.73	0.24	3.07**	0.74	0.25	3.00**	0.75	0.25	3.07**
Networking							0.15	0.05	3.17**		
Positive affect										0.43	0.09	5.11**
Δ Deviance					0.96			198.69**			
Level 1 intercept		0.8654			0.8651			0.8224			0.7808
Level 2 intercept		1.3820			1.3333			1.3401			1.3486

a *Gender (male = 1; female = 2)*.

b *Education (1 = bachelor degree; 2 = master degree; 3 = Ph.D.; 4 = habilitation)*.

** *p < 0.01*.

### Daily networking and career-related outcomes

#### Career optimism

In Hypothesis 3, we predicted that day-level networking would be positively related to career optimism at bedtime. Results are shown in Table 4. Analyses showed that Model 1, in which the control variables were entered, did not show an improvement over the null model (Δ – 2 × log = 1.12, ns). Model 2, which also included networking, showed a significant improvement over Model 1 (Δ −2 × log = 120.67, *p* < 0.01). Analyses indicated that day-level networking was positively related to career optimism at bedtime (γ = 0.09; *SE* = 0.03, *t* = 2.58, *p* < 0.05). Thus, Hypothesis 3 was confirmed.

**Table 4:** Multilevel estimates for models predicting day-specific career optimism at bedtime.

**Variable**	**Null model**			**Model 1**			**Model 2**			**Model 3**		
	**Estimate**	***SE***	***t***	**Estimate**	***SE***	***t***	**Estimate**	***SE***	***t***	**Estimate**	***SE***	***t***
Intercept	3.22	0.07	48.92**	3.20	0.06	51.34**	3.22	0.07	49.46**	3.22	0.07	49.46**
Age				−0.01	0.02	−0.39	−0.01	0.02	−0.45	−0.01	0.02	−0.44
Gender^a^				0.03	0.13	0.23	−0.02	0.14	−0.15	0.01	0.13	0.10
Tenure				−0.02	0.02	−0.73	−0.01	0.02	−0.21	−0.00	0.02	−0.17
Education^b^				0.04	0.14	0.26	−0.42	0.15	−0.29	−0.03	0.15	−0.18
Trait networking				0.09	0.14	0.67	0.10	0.14	0.71	0.11	0.14	0.79
Trait positive affect				0.55	0.13	4.29**	0.53	0.13	3.99**	0.51	0.13	3.83**
Networking							0.09	0.03	2.58*		
Positive affect										0.25	0.06	4.13**
Δ Deviance					1.12			120.67**			
Level 1 intercept		0.5873			0.5867			0.5805			0.5559
Level 2 intercept		0.7245			0.6763			0.6756			0.6831

a *Gender (male = 1; female = 2)*.

b *Education (1 = bachelor degree; 2 = master degree; 3 = Ph.D.; 4 = habilitation)*.

* *p < 0.05*.

** *p < 0.01*.

#### Career satisfaction

In Hypothesis 4, we predicted that day-level networking would be positively related to career satisfaction at bedtime. Results are shown in Table 5. Analyses showed that Model 1, in which the control variables were entered, did not show an improvement over the null model (Δ −2 × log = 3.83, ns). Model 2, which also included networking, showed a significant improvement over Model 1 (Δ −2 × log = 106.48, *p* < 0.01). Analyses indicated that day-level networking was positively related to career satisfaction at bedtime (γ = 0.08; *SE* = 0.03, *t* = 2.46, *p* < 0.05). Thus, Hypothesis 4 was confirmed.

**Table 5:** Multilevel estimates for models predicting day-specific career satisfaction at bedtime.

**Variable**	**Null model**			**Model 1**			**Model 2**			**Model 3**		
	**Estimate**	***SE***	***t***	**Estimate**	***SE***	***t***	**Estimate**	***SE***	***t***	**Estimate**	***SE***	***t***
Intercept	3.45	0.07	51.75**	3.44	0.06	53.42**	3.41	0.07	51.12**	3.41	0.07	51.14**
Age				−0.02	0.02	−1.14	−0.02	0.02	−1.08	−0.02	0.02	−1.03
Gender^a^				0.12	0.13	0.92	0.04	0.14	0.284	0.07	0.14	0.51
Tenure				0.00	0.02	0.15	0.00	0.02	0.20	0.00	0.02	0.14
Education^b^				0.06	0.15	0.44	0.01	0.15	0.09	0.03	0.15	0.22
Trait networking				−0.02	0.14	−0.128	−0.03	0.15	−0.22	−0.04	0.15	−0.30
Trait positive affect				0.48	0.13	3.64**	0.48	0.14	5.50**	0.45	0.14	3.29**
Networking							0.08	0.03	2.46*		
Positive affect										0.15	0.06	2.48*
Δ Deviance					3.83			106.48**			
Level 1 intercept		0.5512			0.5507			0.5572			0.5424
Level 2 intercept		0.7455			0.7128			0.7027			0.7062

a *Gender (male = 1; female = 2)*.

b *Education (1 = bachelor degree; 2 = master degree; 3 = Ph.D.; 4 = habilitation)*.

* *p < 0.05*.

** p < 0.01

### The mediating role of pa in the relationship between networking and its short-term consequences

As our hypotheses refer to low level mediation (Kenny et al., 2003) or the 1-1-1 model (Krull and MacKinnon, 2001) and our models do not contain random effects for the independent variables or the mediator, we used a Sobel test to examine mediation. This procedure essentially tests the significance of the mediated path *ab*, that refers to the product of the paths from the independent variable to the mediator (i.e., path *a*) and the subsequent path from the mediator to the dependent variable (path *b*) when controlling for the independent variable.

For all four mediation hypotheses, path *a* refers to the effect of networking on positive affect (see Table 6). To establish this path, we first ran a null model with positive affect as the outcome and the intercept as the only predictor variable. In Model 1, we included the control variables (i.e., age, gender, tenure, education, trait networking, and trait positive affect). In Model 2, daily networking was added as the relevant predictor variable. Step 1 was supported, as day-level networking was positively related to PA (γ = 0.10*; SE* = 0.03*, t* = 2.88*, p* < 0.01). Coefficients for path *b* are depicted in Table 7, where we show models with control variables, the independent variable (networking), the mediator (positive affect), and the four dependent variable as outlined in Hypotheses 5a−5c.

**Table 6:** Multilevel estimates for models predicting day-specific positive affect at the end of work.

**Variable**	**Null model**			**Model 1**			**Model 2**		
	**Estimate**	***SE***	***t***	**Estimate**	***SE***	***t***	**Estimate**	***SE***	***t***
Intercept	2.77	0.05	53.64**	2.77	0.05	55.77**	2.77	0.05	55.82**
Age				−0.01	0.01	−0.45	−0.01	0.01	−0.48
Gender^a^				−0.07	0.10	−0.71	−0.04	0.10	−0.38
Tenure				−0.00	0.02	−0.27	−0.00	0.02	−0.26
Education^b^				0.20	0.11	1.81	0.21	0.11	1.92
Trait networking				−0.01	0.11	−0.09	−0.01	0.11	−0.12
Trait positive affect				0.35	0.10	3.51**	0.35	0.10	3.54**
Networking							0.10	0.03	2.88**
Δ Deviance					6.20			14.69**
Level 1 intercept		0.5772			0.5767			0.5375
Level 2 intercept		0.5541			0.5256			0.5336

a *Gender (male = 1; female = 2)*.

b *Education (1 = bachelor degree; 2 = master degree; 3 = Ph.D.; 4 = habilitation)*.

** *p < 0.01*.

**Table 7:** Multilevel estimates for models testing the mediating role of positive affect in relationship between networking at the end of work dependent variables reported at bedtime.

**Variable**	**Emotional exhaustion**			**Job satisfaction**			**Career optimism**			**Career satisfaction**		
	**Estimate**	***SE***	***t***	**Estimate**	***SE***	***t***	**Estimate**	***SE***	***t***	**Estimate**	***SE***	***t***
Intercept	2.18	0.04	57.60**	5.18	0.12	42.70**	3.23	0.07	49.50**	3.41	0.07	51.17**
Age	−0.02	0.01	−2.01*	0.02	0.04	0.56	−0.01	0.02	−0.47	−0.02	0.02	−1.06
Gender^a^	0.04	0.08	0.57	−0.02	0.25	−0.06	0.00	0.13	0.02	0.07	0.14	0.50
Tenure	0.00	0.01	0.10	0.01	0.04	0.23	−0.00	0.02	−0.15	0.00	0.02	0.20
Education^b^	−0.02	0.08	−0.26	0.13	0.27	0.50	−0.03	0.14	−0.19	0.02	0.15	0.15
Trait networking	0.20	0.08	2.42*	−0.47	0.27	−1.72	0.10	0.14	0.68	−0.05	0.15	−0.32
Trait positive affect	−0.13	0.08	−1.66	0.76	0.25	3.09**	0.51	0.13	3.85**	0.46	0.14	3.37**
Networking	−0.02	0.03	−0.72	0.10	0.05	2.20*	0.06	0.03	1.68	0.06	0.03	1.93
Positive affect	−0.37	0.05	−6.98**	0.41	0.08	4.90**	0.24	0.06	3.97**	0.13	0.06	2.23*
Δ Deviance		73.66**^c^			1236.53**			18.26**			5.18
Level 1 intercept		0.4218			0.7777			0.5503			0.2874
Level 2 intercept		0.3701			1.3493			0.6841			0.5005

a *Gender (male = 1; female = 2)*.

b *Education (1 = bachelor degree; 2 = master degree; 3 = Ph.D.; 4 = habilitation)*.

c *Deviance comparison with the respective Model 2 (networking as the only Level 1 predictor)*.

* *p < 0.05*.

** *p < 0.01*.

For Hypothesis 5a, predicting that positive affect mediated the effect of networking on emotional exhaustion, path b, when controlling for networking, was also significant (γ = −0.37, *SE* = 0.05, *t* = −6.98, *p* < 0.001; see Table 7), yielding an indirect effect of *ab* = −0.037, *z* = −3.04, *p* < 0.01. Hypothesis 5a was thus supported. Hypothesis 5b predicted that the effect of networking on job satisfaction would be mediated by positive affect. Table 7 shows that the effect of positive affect on job satisfaction (i.e., path *b*) was significant (γ = 0.41, *SE* = 0.08, *t* = 4.90, *p* < 0.001). The Sobel test indicated that the product was also significant with *ab* = 0.043, *z* = 2.79, *p* < 0.01. Thus, Hypothesis 5b was also confirmed.

Next, we examined hypothesis 5c, whether positive affect mediated the relationship between networking and career optimism. In addition to path *a* described two paragraphs above, the relationship between positive affect and career optimism (path *b*) was also significant (γ = 0.24, *SE* = 0.06, *t* = 3.97, *p* < 0.001; see Table 7). The product of the two paths was also significant, *ab* = 0.025, *z* = 2.56, *p* < 0.05, confirming Hypothesis 5c. In Hypothesis 5d, we predicted that positive affect mediates the relationship between networking and career satisfaction. Table 7 shows that positive affect was significantly related to career satisfaction (γ = 0.13, *SE* = 0.06, *t* = 2.23, *p* < 0.05). However, the product of the two path coefficients was only marginally significant (*ab* = 0.013, *z* = 1.81, *p* = 0.069). Thus, we only found marginal support for Hypothesis 5d.

## Discussion

Going beyond the long-term benefits of networking described in the literature (Wolff and Moser, 2010; Blickle et al., 2012; Ng and Feldman, 2014a,b), this is the first study to investigate proximal consequences of daily networking behavior. We use a within-person perspective to emphasize that variations in daily networking also have positive short-term effects on employees' daily job and career-related evaluations as well as well-being. Daily networking is a means to proactively acquire valued resources in the work domain and might even trigger gain spirals. In addition, our findings show that even though scholars depict networking as a rather stable behavior syndrome, there exists substantive variation in the level of daily networking behaviors. Note however, that while within-person differences do exist, our “classical” trait-like between-person networking measure does still explain significant differences in daily networking. Both, the within-person and the between-person perspective are important in networking research, because next to daily fluctuations of networking behaviors, individuals also differ in their respective amount of dispositional networking behavior.

Our findings extend networking research by supporting Conservations of Resources' (COR; Hobfoll, 2001, 2011) theoretical account of resource gain in the workplace context (Gorgievski et al., 2011). We believe that COR theory and our delineation of the resource building processes of networking provide further insights into how networking leads to beneficial long-term outcomes. In line with our assumption that networking is part of a gain spiral, we find that within-person differences in daily networking behaviors yield positive outcomes for a range of variables. We consider this finding to be important as it shows that investments in networking behaviors pay off in the short and long run. A plausible alternative would have been that daily investments in networking deplete daily resources and result in exhaustion, although it has positive consequences in the long run. In contrast, our study shows that daily benefits of networking appear to outweigh the time and effort that people invest in networking.

While between-person studies have shown relations between networking and satisfaction measures, we also emphasize that networking across the day is important to other central variables of COR theory. For instance, networking during the workday reduces feelings of exhaustion in the evening. This finding is of particular importance as it provides further support for the basic assumption that networking is a means to acquire resources which in turn facilitate daily work behavior. By this means, networking might function as a buffer in stressor-strain relationships and might not only yield positive outcomes, but also protect people from negative ones. In addition, the relationship of daily networking with daily career optimism shows that networking is a means to build key resources. Increases in daily career optimism should affect how people tackle subsequent work or career challenges and this finding provides evidence for the initiation of gain spirals by networking.

We further found that positive affect mediated the association between daily networking behavior, well-being, job attitudes as well as career-related outcomes (though the mediating effect on career satisfaction was only marginal). In line with other studies, this corroborates the importance of affective or emotional states in the relationship between daily resources and outcomes (Xanthopoulou et al., 2012). In line with the broaden-and-build theory (Fredrickson, 2001, 2003), networking episodes are associated with positive emotional experiences at work, which in turn affect employees' well-being as well as job and career related evaluations. This implies that not only specific resource gains themselves are beneficial in the context of networking but also that daily networking helps employees to experience immediate positive reactions which in turn are linked with resources. Moreover, employees might anticipate future resource gains, resulting in positive feelings. We presume that positive affect is caused by success in resource acquisition, its subsequent deployment, and also by the experience of positive social interactions while networking. In sum, the present study further helps to understand *why* networking is beneficial for acquiring resources. Other potential mediators (e.g., self-efficacy beliefs, co-worker support) should be investigated in future research.

The present study further highlights that time and research focus play an important role regarding consequences of networking. In this regard, we provide important insights into daily networking. Yet, we might have unraveled more questions than we answered. First, although we could show that networking has positive consequences from a daily perspective, this does not mean that investments in networking come at no cost at all. Future research using even finer-grained time frames (e.g., experimental studies or experience sampling studies with smaller-grained time frames) might find evidence for immediate costs and provide further insights into the micro processes involved in networking (Wingender and Wolff, 2016). Second, in contrast to the negative (i.e., attenuating) relationship between daily networking and exhaustion, our between-person measure of networking is positively associated with exhaustion (cf. Table 2). While we did not theorize about such differences, this finding shows that within-person differences in daily networking and between-person differences in networking levels do not need to have the same direction necessarily. While we contribute to the literature by highlighting daily processes and short-term consequences of networking behavior, future research needs to further examine the consequences and relationships of long-term effects. We believe that, for instance, cross-level effects and personality traits as potential moderators of these outcomes would be particularly viable.

With regard to practical implications, our findings suggest that organizations and HR managers are well-advised to create organizational environments which enable and stimulate networking behaviors. These might include office arrangements (e.g., informal meeting opportunities at the vending machine) and also work design (e.g., taking time for networking into account when planning workshops). So far, research has focused on long term individual outcomes of networking which are potentially beneficial (e.g., job performance), but also might be detrimental to organizations such as job moves and the associated loss of human capital (Porter et al., 2016). Yet, in the short run, organizations might also benefit from fostering networking behavior as it helps employees to conserve their resources on a daily level. Employees who network exhibit more positive attitudinal outcomes and less exhaustion, a combination that might potentially help employees to fulfill their work tasks. Given that networking also improves positive affect, employee networking might also contribute to a more positive climate within an organization. In addition our findings may prove valuable to those who want to motivate people to network, such as supervisors, trainers, or coaches. Often people indicate they abstain from networking because it depletes resources (Bensaou et al., 2013). Though acknowledging the immediate depletion of resources (Wingender and Wolff, 2016), people should be aware that the detrimental effects are rather short lived and, as we show, networking expands its positive effects even into employees' private lives. Although networking *per se* is time-consuming (e.g., offering help to others in work-related matters, going out with others for drinks after work), it reduces emotional exhaustion and increases job satisfaction, or simply makes employees who network feel better at the end of their workday. Though people may know that networking is beneficial in the long run, the present findings might be well-suited to encourage employees or trainees to network by providing additional information on short term expectancies about networking outcomes (Vroom, 1964; Porter and Woo, 2015). In this vein, the present study reiterates that employees are well-advised to network—not only with regard to long-term outcomes, but also from a daily perspective.

### Limitations

The present study has limitations that should be noted. First, we solely collected data from academic staff at German universities which can restrict the generalizability of our findings. Although we do not assume that our sample differs in main aspects (e.g., networking) from others, findings should be replicated with samples from different occupational fields (e.g., blue-collar workers) and different cultures (e.g., collectivistic cultures) in order to evaluate whether there exist differences in networking behaviors and its respective outcomes. Second, building on COR theory (Hobfoll, 2001, 2011), we focused on specific short-term reactions in terms of well-being, job attitudes and career-related outcomes. Although our theorizing incorporated assumptions on resources attained by networking, apart from optimism, we did not directly measure other resources, such as receiving strategic information or having someone put in a word for a person. However, this is an accepted practice in research on the COR, as resources might be highly idiosyncratic (Halbesleben et al., 2014). Moreover, this practice also has some methodological advantages. Because it would be most viable to assess the resources people have acquired at the end of the workday, the relatedness of networking and resources poses the threat of inflated associations by means of common measurement occasion. By assessing outcomes at bedtime, we were able to temporally separate the assessment of networking and outcomes. Third, we emphasize that our focus on prominent outcomes of daily networking according to COR theory is by no means exhaustive. We do not attempt to explain antecedents of differences in daily networking. The variance in daily networking behaviors highlights that networking is contingent upon daily differences in other variables. For instance, work tasks might explain why “networkers” show less networking behaviors on some days (e.g., when they grade an exam) and more on other days (e.g., at a conference). In addition, our outcomes focus on constructs from work and career domains. Future research should expand the focus and investigate effects of daily networking on the work-life interface, for instance, recovery experiences at bedtime, or work-family conflict and enrichment. Finally, constructs were measured via self-report which may be partially susceptible to common method bias. Yet, our daily design with two separate measurement points should have reduced common method bias. Nonetheless, future research should include supervisor- or peer ratings and observational data.

## Conclusion

In conclusion, the present research clearly shows that networking is worth studying from a finer-grained, within-person perspective. Beyond numerous positive long-term effects of networking (e.g., salary, promotions), daily networking relates to employees' immediate affective experiences and resource gains. Results of the present study integrate networking research (Wolff and Moser, 2006; e.g., Ng and Feldman, 2014a,b) with a positive organizational behavior perspective (Quick et al., 2010). Organizations and employees are well-advised to engage in daily networking behavior as it seems to trigger a resource gain spiral.

## Data availability statement

The raw data supporting the conclusions of this manuscript will be made available by the authors, without undue reservation, to any qualified researcher.

## Ethics statement

This study was carried out in accordance to the APA ethical guidelines. Participants were informed about the study aim, duration, and how to obtain information on results. They were informed about whom to contact for any questions regarding research or their rights, about their right to leave the survey at any time without any prejudice, and subjects were guaranteed anonymity and confidentiality of their data. Ethical review and approval was not required for this study as per the institutional and national requirements.

## Author contributions

JV and H-GW were responsible for participant recruitment. JV also for assessment and data analyses. JV wrote the first draft of this paper and then both authors iteratively revised this version.

### Conflict of interest statement

The authors declare that the research was conducted in the absence of any commercial or financial relationships that could be construed as a potential conflict of interest.
